# Recognition of Pancreatic Insufficiency

**Published:** 1917-09

**Authors:** 


### ABSTRACTS

**Have you ever felt that this department of the BUFFALO MEDICAL JOURNAL was lacking in abstracts of your specialty, or that such as were published lacked the expert judgement which you yourself could have exercised in selection and preparation? If not, you are more charitable than the editor has been to himself. If so, will you assist in abstracting from a few journals, and thus make this department more representative and more valuable? Incidentally, there are few forms of study more helpful to the worker than this.**

---

**Recognition of Pancreatic Insufficiency.** H. Ryerson Decker, Pittsburgh, Boston M. & S. Jour., June 21. There is no pathognomonic symptomatology and even inspection at operation does not reveal the finer lesions. The oil test breakfast of Boldyreff and Volhard tends to cause regurgitation into the stomach and hence to allow tests for tryptic digestion. 200-250 c.c. of olive oil or cream is given plus milk of magnesia (Lewinski) to neutralize acidity. The test meal is not tolerated by all, it does not invariably cause regurgitation of duodenal contents, HCI may be secreted and thus inhibit the tryptic test (and conversely, without any organic or marked functional defect, pancreatic juice may not be secreted at the particular time. Ed.) Einhorn's method of duodenal intubation is theoretically ideal but many candidly confess practical difficulties and others emphasize the fact that, without abnormality, pancreatic secretion may not be called into action. Sahli's glutoid capsule test, theoretically limited to the digestion of the capsule by pancreatic secretion alone and liberating some rapidly eliminated substance to be tested for in the urine or saliva, has not given dependable results in the hands of others. Schmidt's cell nuclei test postulates that the nuclei are digested solely by pancreatic nucleases. Various sources of nuclei are employed, alcohol-hardened beef, thymus gland, red blood cells of fowls, etc., and identification is aided by inclusion in silk or gauze capsules, attachment to beads (Einhorn), accompaniment by indicators (barium sulphate, Fronzig). It is doubtful whether nucleases are derived solely from the pancreas and it is conceded that more than 36 hours' delay in the intestine allows bacterial decomposition while, on the other hand, at least 6 hours' sojourn is necessary. Azotorrhoea is subject to similar criticisms. Normally the faeces contain 5-10% of the intake and the presence of 30% or more naturally suggests pancreatic failure. Creatorrhoea, the presence of an excess of muscle fibres, say more than 2 in an average field on a diet of 2 ounces of meat a day; tryptic power; steatorrhoea; presence or absence of lipase and diastase in the faeces; lecithin, normally ½ gram a day in the faeces, destroyed by pancreatic secretion but absorbed, even if thus in excess by bile; are all subject to both explainable and unexplainable variations.

All the foregoing tests are of external secretion and none is dependable by itself. Of tests of internal secretion, may be mentioned glycosuria but only about 30% of glycosuric cases are of pancreatic origin. The Cammidge reaction is now held by most investigators, not only to be lacking in pathognomonic value but, even on theoretic grounds, to be an index of general disturbance of carbohydrate metabolism and not limited to the pancreatic function. Wohlgemut, 1908, apparently demonstrated that an excess of diastase in the urine is indicative of pancreatic lesions but the author has found such excess in appendicitis and ovarian cyst and low readings for diastase in cancer of the head of the pancreas. Lowi. 1908, found that adrenalin, 1: 1000 caused no mydriasis in normal animals but did when the pancreas had been removed. Corresponding reactions were noted clinically in human beings, subject to the usual variations and exceptions. The author found mydriasis in 18 of 500 cases but only 2 were known to have a pancreatic lesion. However, 3 of the positive reactions occurred among 15 gall bladder cases and it is well known that there is a degree of association of biliary and pancreatic lesions. In 6 of the 18 positive tests, pancreatic lesion could not be definitely excluded.

(Note: 30 years ago, the best clinical teachers emphasized the difficulty of diagnosis of pancreatic disease. It remains true that no single, easy test exists, not even easy in the sense that a procedure can be referred by the clinician to a laboratory worker. On the other hand, routine faecal examinations, even of comparatively simple nature, in connection with the studies of diet and nutrition, and the usual physical examinations, carefully applied to the individual case, enable us to claim a fairly efficient diagnostic ability).

